# Vitamin A deficiency impairs the immune response to intranasal vaccination and RSV infection in neonatal calves

**DOI:** 10.1038/s41598-019-51684-x

**Published:** 2019-10-22

**Authors:** Jodi L. McGill, Sean M. Kelly, Mariana Guerra-Maupome, Emma Winkley, Jamie Henningson, Balaji Narasimhan, Randy E. Sacco

**Affiliations:** 1 Department of Veterinary Microbiology and Preventative Medicine, Iowa State University, Ames, IA USA; 2 Department of Chemical and Biological Engineering, Iowa State University, Ames, IA USA; 3 Department of Diagnostic Medicine and Pathobiology, Kansas State University, Manhattan, KS USA; 4 Ruminant Diseases and Immunology Research Unit, National Animal Disease Center, Agricultural Research Service, USDA, Ames, IA USA; 5 Nanovaccine Institute, Iowa State University, Ames, IA USA

**Keywords:** Protein vaccines, Viral infection

## Abstract

Respiratory syncytial virus (RSV) infection is a leading cause of severe acute lower respiratory tract infection in infants and children worldwide. Vitamin A deficiency (VAD) is one of the most prevalent nutrition-related health problems in the world and is a significant risk factor in the development of severe respiratory infections in infants and young children. Bovine RSV (BRSV) is a primary cause of lower respiratory tract disease in young cattle. The calf model of BRSV infection is useful to understand the immune response to human RSV infection. We have previously developed an amphiphilic polyanhydride nanoparticle (NP)-based vaccine (i.e., nanovaccine) encapsulating the fusion and attachment proteins from BRSV (BRSV-NP). Calves receiving a single, intranasal dose of the BRSV-NP vaccine are partially protected from BRSV challenge. Here, we evaluated the impact of VAD on the immune response to the BRSV-NP vaccine and subsequent challenge with BRSV. Our results show that VAD calves are unable to respond to the mucosal BRSV-NP vaccine, are afforded no protection from BRSV challenge and have significant abnormalities in the inflammatory response in the infected lung. We further show that acute BRSV infection negatively impacts serum and liver retinol, rendering even well-nourished individuals susceptible to VAD. Our results support the use of the calf model for elucidating the impact of nutritional status on mucosal immunity and respiratory viral infection in infants and underline the importance of VA in regulating immunity in the respiratory mucosa.

## Introduction

Vitamin A deficiency (VAD) is one of the most common nutritional disorders in the world. An estimated 250 million preschool-aged children are VAD, with at least 5 million showing clinical signs^1^. VAD is associated with increased susceptibility to disease, poor response to vaccination and increased mortality^2,3^. Although frank VAD is generally considered a disease of the developing world, marginal VAD can be problematic even in developed countries such as the U.S.^1,4–6^. Over 20% of the population in the developed world does not reach the recommended intake, and has plasma and liver concentrations of vitamin A lower than those accepted as normal^4,5^. Premature infants are born VAD, and despite supplementation, can remain biochemically deficient for months to years after birth^7,8^. Even at subclinical levels, VAD has been linked to increased incidence and severity of respiratory diseases^4,5,9^.

Vitamin A refers to a group of fat-soluble retinoids, including retinol, retinal and retinyl esters. Animals are incapable of de novo VA synthesis; therefore, dietary VA is obtained in the diet as preformed VA from animal sources, or as provitamin carotenoids such as beta-carotene from plant sources. VA is stored in the liver, and when needed, is released into the blood stream in the form of retinol. Retinol is converted to its bioactive form, retinoic acid (RA), by a series of enzymes including alcohol dehydrogenases and retinaldehyde dehydrogenases. VA and RA play complex roles in regulating the immune system. VAD causes reduced or altered T and B cell responses to a number of pathogens^10–15^; dysregulated production of type I IFN^16^; and alterations in numbers and functions of innate immune populations including monocytes, NK cells, and dendritic cell subsets^16,17^. The roles of VA and RA have been studied in the context of immunity in the gastrointestinal tract. RA plays an important role in regulating the homing of T cells to the gut^18^, and in promoting the development of FoxP3^+^ regulatory T cells^19^. Less is known about the importance of VA and RA in lung immune function. Given the known epidemiologic link between VAD and respiratory disease, there is a need to define the role of VA and RA immunity in the respiratory tract.

Human respiratory syncytial virus (RSV) is a leading cause of severe acute lower respiratory tract disease in infants and young children worldwide^20,21^ and accounts for up to 70% of hospitalized bronchiolitis cases in industrialized countries^22^. Globally, there are an estimated 33 million new episodes of HRSV-associated disease in children under 5 years of age with more than 100,000 resultant deaths^23^. Severe RSV infection has been linked with the development and exacerbation of recurrent wheezing and asthma^24^, and is a predisposing factor to the development of otitis media^25^. Despite the high burden, treatment for RSV infection is largely supportive, and there is no vaccine available to prevent or reduce disease.

Bovine respiratory syncytial virus (BRSV) is closely related to human RSV and is a primary cause of severe acute lower respiratory tract disease in young cattle. BRSV infection in calves is strikingly similar to RSV infection in humans, with similarities in age dependence, innate and adaptive immune responses, and disease pathogenesis^26–28^. The calf model is useful to understand the immune response to RSV infection because it is a physiologic host-pathogen interaction, represents a tractable model of the infant immune system, and is a scalable model which can be used to test novel therapeutics and intervention strategies.

Nanoparticles (NP) composed of biodegradable polyanhydrides have shown impressive versatility and immunogenicity as carriers for vaccine antigens^29–33^. We have recently published on the development of an amphiphilic polyanhydride copolymer NP based vaccine (i.e. nanovaccine) comprised of a 50:50 molar composition of 1,8-bis(*p*-carboxyphenoxy)-3,6-dioxaoctane (CPTEG) and 1,6-bis(*p*-carboxyphenoxy)hexane (CPH), encapsulating the fusion (F) and attachment (G) proteins from BRSV (BRSV-NP)^34^. The BRSV-NP vaccine exhibited sustained release kinetics of antigen *in vitro* and maintained the immunogenicity of the antigen payload. Calves receiving a single, intranasal dose of the BRSV-NP vaccine were partially protected from BRSV challenge, with reduced viral loads in the lung, reduced virus shedding and significantly reduced lung pathology compared to their unvaccinated cohorts^34^. In this study, protection in calves was associated with the induction of virus-specific IgA responses in nasal secretions and bronchoalveolar lavage fluid, and virus-specific cellular immune responses in the lower airways and peripheral blood^34^.

Given the high burden of RSV disease in both humans and animals, development of a safe and effective vaccine is a critical goal. Importantly, however, a vaccine is only half of the equation and the status of the host immune system has a profound impact on vaccine efficacy, and ultimately, disease susceptibility. Understanding the factors that may negatively affect the efficacy of vaccines in target populations is also vital for an effective immunization program. VAD is endemic in the geographical regions which are hit hardest by RSV^1^, and is also highly prevalent in premature infants, a population known to be at increased risk from RSV^7,8^. Epidemiologically, there is significant correlation between VAD and increased susceptibility to and severity of RSV infection^35,36^; however, the impact of the deficiency on mucosal immune function has not been explored in this context experimentally. To this end, we generated a calf model of VAD, assessed the immune response to mucosal BRSV-NP vaccination and subsequent BRSV challenge, and compared the responses to VA sufficient (VAS) calves. Here, we report that while VAS, BRSV-NP immunized calves are protected from severe RSV-associated disease, VAD calves fail to respond to intranasal BRSV-NP vaccination and develop severe BRSV-associated disease. VAD, BRSV-NP immunized calves do not mount an IgA response in the respiratory tract, nor do they generate virus-specific T cell responses in the lungs or peripheral blood. Gene expression studies demonstrated that VAD calves present with significant abnormalities in the inflammatory milieu in the infected lung, with alterations in Th1 and Th17 immune responses, and impaired mucin production. We further show that acute respiratory viral infection has a significant negative impact on circulating and stored VA levels, causing even vitamin-replete calves to become VA deficient. Thus, our results show that VA status has a significant impact on the mucosal immune system and resistance to respiratory viral infection.

## Results

### Serum and liver retinol levels

**Figure 1:**
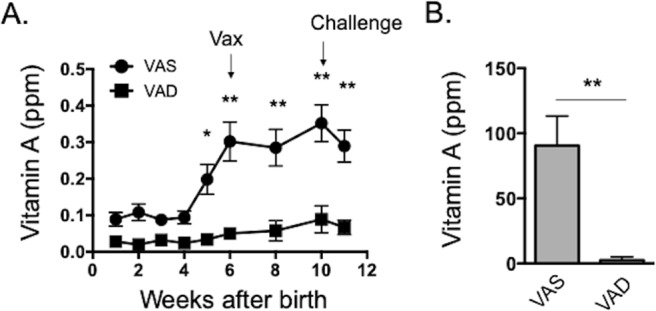
Retinol concentrations in the serum and liver of VAS and VAD calves. Calves were divided into two groups. VAD animals (n = 18) were fed fractionated colostrum replacer, milk replacer and calf starter devoid of VA. VAS animals (n = 19) were fed matched control diets with added VA. (**A**) Serum retinol was measured weekly in calves starting at 1 week of age. Data are presented as mean ± SEM. *p < 0.05, **p < 0.01 by two-way ANOVA and Tukey’s posttest. (**B**) Liver retinol was measured at necropsy, on day 7 after BRSV infection. Data are presented as mean ± SEM. **p < 0.001 by student’s *t*-test.

To determine the impact of VAD on the response to mucosal vaccination and subsequent RSV challenge, we first established two groups of calves with differing levels of serum and liver retinol. Calves are born with low VA levels and colostrum is a major source of VA and other fat-soluble micronutrients^37^. Therefore, all calves received fractionated colostrum replacer with or without VA restored, and were placed on a VAS or VAD milk replacer diet. Serum retinol levels were evaluated weekly, starting after calves were on the differential diets for 1 week. As shown in Fig. 1A, all animals had low serum retinol levels at week 1, but these levels increased in the VAS group, reaching normal serum retinol concentrations by 5–6 weeks of age. The normal range for serum retinol in juvenile calves (30–300 days) is 0.25–0.33 ppm^38^. Plasma VA levels are tightly regulated by the liver, and therefore not optimal for determining VA status. To confirm VA status in our two treatment groups, liver samples were collected at the time of necropsy. The normal range for liver retinol in juvenile calves is 75–130 ppm^38^. As seen in Fig. 1B, calves in the VAD treatment group had below normal retinol stores in the liver at the time of necropsy, while VAS calves had normal liver stores.

### VAD calves are not protected by intranasal BRSV-F/G nanoparticle vaccination

**Figure 2:**
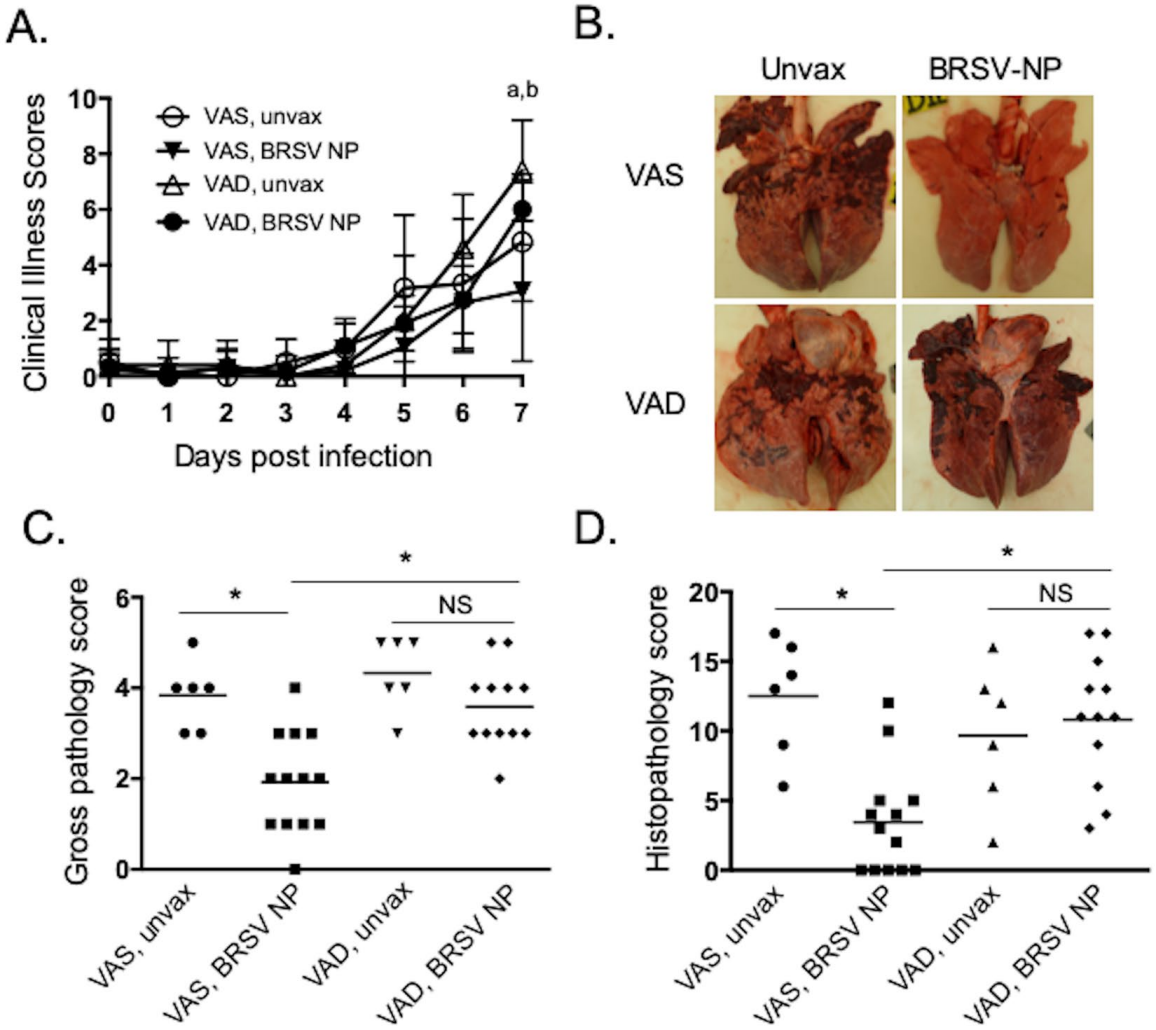
VAD calves are not protected by mucosal BRSV-NP vaccination. VAS (n = 13) and VAD (n = 12) calves, aged 5 weeks, were vaccinated with a single, i.n. dose of the BRSV-NP vaccine. Age-matched VAS (n = 6) and VAD (n = 6) control calves remained unvaccinated. Four weeks after vaccination, all calves were challenged via aerosol inoculation with 10^4^ TCID_50_ BRSV strain 375. (**A**) Calves in all four groups were monitored daily and assigned a clinical score using the criteria outlined in Materials and Methods. Data represent means ± SEM. ^a^p < 0.05 between VAS/BRSV-NP vaccinated and VAS, unvaccinated calves; ^b^p < 0.01 between VAS/BRSV-NP vaccinated and VAD/BRSV-NP vaccinated. Results were analyzed by 2-way ANOVA with repeated measures, followed by Tukey’s multiple comparisons test. (**B,C**) Animals were humanely euthanized on day 7 post infection. The extent of gross pneumonic consolidation was evaluated based upon the percent of lung affected (0 = free of lesions; 1 = 1–5% affected; 2 = 5–15% affected; 3 = 15–30% affected; 4 = 30–50% affected; 5 = > 50% affected). Representative images of lungs from one animal in each group are shown in (**B**). Top panel: VAS calves (with or without BRSV-NP immunization). Bottom panel: VAD calves (with or without BRSV-NP immunization). Aggregate gross pathology results from all groups and all animals are depicted in (**C**). *p < 0.05 as determined by Kruskal-Wallis test, followed by Dunn’s multiple comparisons test. NS = not significant. Sections of affected and unaffected lung were collected from multiple locations and microscopic lesions were evaluated by a pathologist in a blinded manner using a scoring system we have previously described (see Materials and Methods). Aggregate histopathology scores from all animals are depicted in (**D**). *p < 0.05 as determined by Kruskal-Wallis test, followed by Dunn’s multiple comparisons test. NS = not significant.

We have previously demonstrated that the intranasal BRSV-F/G nanovaccine induces partial protection from BRSV challenge in young, colostrum-replete calves^34^. To determine the impact of VAD on the response to intranasal vaccination, 13 calves from the VAS treatment group and 12 calves from the VAD treatment group were immunized with the BRSV-NP vaccine. In prior studies, inclusion of soluble antigen with CPTEG:CPH NP-encapsulated protein has been used to induce rapid immunogenicity of the nanovaccines^29,39^. Therefore, in this study we also sought to determine whether inclusion of soluble F/G protein improves the efficacy of the BRSV-NP vaccine. Calves were immunized intranasally with a single dose of 145 mg BRSV-F/G NP (encapsulating ~1 mg each of the BRSV F and G proteins), co-administered with 1 mg each of soluble F and G proteins in 5 mL of sterile saline. Six VAS calves and six VAD calves served as unvaccinated controls. Four weeks after immunization, calves were challenged via aerosol inoculation with ~10^4^ TCID_50_ BRSV strain 375. Calves were monitored daily for clinical signs, including body temperature, appetite, nasal discharge and respiratory effort. Starting on days 4–5 after infection, animals developed fevers and clinical signs of lower respiratory tract disease, including coughing, increased respiration rates and increased respiratory effort. As seen in Fig. 2A, on day 7 post infection, clinical disease was significantly reduced in VAS/BRSV-NP vaccinated calves compared to VAS/unvaccinated animals and compared to VAD/BRSV-NP vaccinated calves. There were no significant differences in the clinical signs observed between VAS/unvaccinated controls, VAD/unvaccinated controls and VAD/BRSV-NP vaccinated calves.

The animals were euthanized on day 7 p.i. and gross lung pathology was evaluated at necropsy. Both VAS and VAD unvaccinated control calves developed lung lesions that were consistent with our previous studies, with bilateral, multifocal to coalescing areas of pneumonic consolidation (Fig. 2B,C). However, as observed in our prior study, VAS/BRSV-NP vaccinated animals were protected from severe disease and exhibited a significant reduction in gross lung pathology (Fig. 2B,C). Surprisingly, however, VAD/BRSV-NP vaccinated calves were not protected by vaccination and developed lung lesions that were similar to the unvaccinated control calves (Fig. 2B,C). Samples of affected and unaffected lung tissue from each animal were evaluated for microscopic lesions using the histopathology scoring system we have previously described^34^. The lesions were consistent with experimental BRSV infection^27,34,40^ with bronchointerstitial pneumonia and necrotizing bronchiolitis. As seen in Fig. 2D, VAS/BRSV-NP vaccinated calves showed significantly reduced histological lesions compared to VAS/unvaccinated calves and VAD/BRSV-NP vaccinated calves. No differences were observed between VAD/unvaccinated and VAD/BRSV-NP vaccinated calves.

Our results corroborate our prior study showing that a single dose of the intranasal BRSV-NP vaccine induces partial protection from BRSV infection in juvenile calves. We observed no benefit from the inclusion of the soluble F/G protein in the BRSV-NP vaccine, as the disease sparing in VAS vaccinated calves was similar to that observed in our prior studies of BRSV-NP vaccinated calves^34^. Our data show that micronutrient status is an important predictor of mucosal vaccine efficacy, as VAD calves that received the BRSV-NP vaccine showed no protection from subsequent BRSV challenge.

### BRSV shedding and lung viral burden

**Figure 3:**
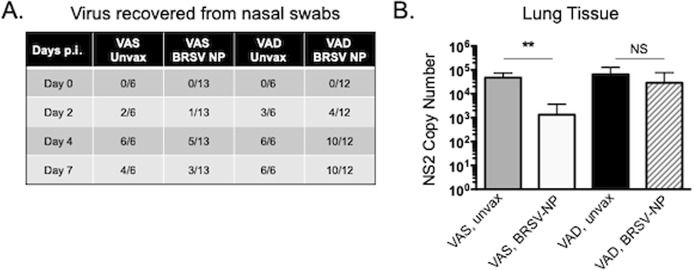
Virus shedding and lung burden in VAS and VAD calves receiving the BRSV-NP vaccine. (**A**) Nasal swab samples were collected on days 0, 2, 4 and 7 after BRSV challenge. Virus isolation was performed on bovine turbinate cells to determine virus shedding. Results are presented as a ratio of BRSV positive samples/total samples. (**B**) Tissue samples were collected from 2–3 representative lesion and non-lesion sites of the lungs on day 7 post-challenge and preserved in RNALater. The RNA was extracted using Trizol reagent. The RNA from multiple sites was then pooled and was analyzed by qPCR for the BRSV NS2 gene. Viral NS2 copy numbers were calculated using standard curves and normalized to the housekeeping gene, S9, to correct for differences in input material. Results represent the mean NS2 copy number of each calf, with n = 6–13 animals/group. The graph depicts means ± SEM of each group. **p < 0.01 as determined by Kruskal-Wallis test, followed by Dunn’s multiple comparisons test.

Nasal swabs were collected from the animals on days 0, 2, 4 and 7 p.i. to detect viral shedding. As seen in Fig. 3A, BRSV was consistently detected in the unvaccinated VAS and VAD controls on all days after infection, and the majority of the VAD/BRSV-NP vaccinated calves. However, virus was detected in the nasal swabs of less than 50% of the VAS/BRSV-NP vaccinated calves at any timepoint after challenge. Lung tissue samples were analyzed for viral burden using qPCR to detect the viral NS2 gene. VAS/BRSV-NP vaccinated calves had significantly reduced lung viral burdens on day 7 p.i. compared to all other groups (Fig. 3B). Consistent with the virus isolation data, VAD/BRSV-NP vaccinated calves had high titers of BRSV in the lungs and showed no reduction in lung viral burden compared to unvaccinated VAD controls.

### VAD calves fail to mount a vaccine-induced mucosal immune response

**Figure 4:**
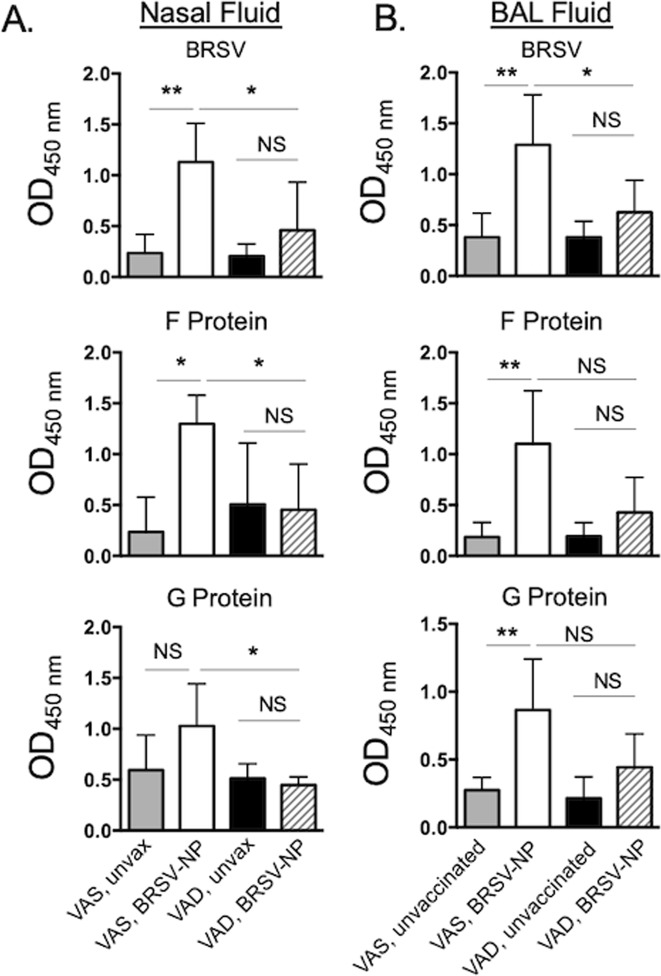
VAD calves fail to produce virus-specific IgA in response to intranasal BRSV-NP vaccination. (**A**) Nasal fluid and (**B**) BAL samples were collected on day 7 post infection. The samples were diluted 1:2 and analyzed by indirect ELISA for BRSV-, F-protein or G-protein specific IgA. Data represent means ± SEM. *p < 0.05 as determined by Kruskal-Wallis test, followed by Dunn’s multiple comparisons test.

We have previously shown that the intranasal BRSV-NP vaccine primes for virus-specific IgA production in the upper and lower respiratory tract, and virus-specific cellular immune responses in the airways and peripheral blood^34^. Given the reduced protection observed in the VAD vaccinated calves, we next examined virus-specific immune responses in the peripheral blood and respiratory tract to BRSV and the F and G proteins. VAS/BRSV-NP vaccinated calves did not mount a significant IgA response to BRSV or the F and G proteins in the nasal fluid prior to BRSV challenge; a result that is consistent with our previous studies. However, on day 7 following BRSV challenge, VAS vaccinated calves had significantly increased concentrations of virus-specific IgA in the nasal fluid (Fig. 4A) and in the BAL (Fig. 4B) compared to unvaccinated VAS calves. This virus-specific IgA response in the respiratory tract included a response to both the F and G proteins (Fig. 4). In contrast to their VAS cohorts, the VAD/BRSV NP vaccinated animals failed to mount a vaccine-induced IgA response in the BAL or nasal fluid (Fig. 4). In ruminants, IgG1 is produced at significant quantities at the mucosal surface; therefore, we also analyzed BRSV-specific IgG (all isotypes) in nasal fluid and BAL fluid on day 7 after infection (Supplementary Fig. 1). Similar to our IgA results, VAS/BRSV NP vaccinated animals had increased levels of virus-specific IgG in the BAL fluid, while reduced quantities of virus-specific IgG were observed in the nasal secretions of unvaccinated animals (both VAS and VAD) and VAD/BRSV NP vaccinated animals. Quantities of virus-specific IgG in the nasal fluid were not significantly different between groups (Supplementary Fig. 1).

Calves in this study were colostrum replete and possessed preexisting BRSV-specific antibody titers. We observed no differences in the virus-specific serum IgG levels between any of the treatment groups in response to vaccination. Serum neutralization titers (mean and range) are depicted for all groups in Table 1.

**Table 1:** BRSV-specific neutralizing antibody titers in serum at baseline and on day 0, prior to BRSV challenge.

Treatment groups	Baseline (prior to vaccination) Mean (range)	Day 0 (prior to BRSV infection) Mean (range)
VAS, Unvax	45.3 (16–64)	29.3 (16–32)
VAS, BRSV NP	50.4 (16–128)	30.77 (16–64)
VAD, Unvax	93.3 (16–256)	45.3 (16–128)
VAD, BRSV NP	45.5 (16–128)	35.7 (8–64)

**Figure 5:**
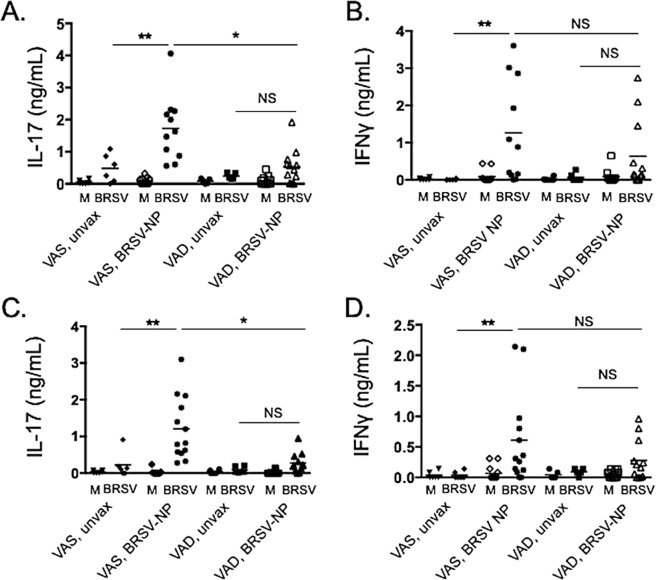
Impaired cellular immune responses in the airways and peripheral blood of VAD/BRSV-NP vaccinated calves. (**A**,**B**) Bronchoalveolar lavage samples were collected during the necropsy on day 7 post infection. BAL cells were stimulated for 72 hours with 0.01 MOI BRSV. Control wells remained unstimulated (M, mock). (C,D) Peripheral blood was collected on day 7 after challenge. PBMC were isolated and stimulated for 6 days with 0.01 MOI BRSV. Control wells remained unstimulated (M, mock). Cell culture supernatants were collected from BAL and PBMC cultures and analyzed by commercial ELISA kit for IL-17 (**A,C**), and IFNγ (**B,D**). Data represent means ± SEM. *p < 0.05, **p < 0.01 as determined by one-way ANOVA and Tukey’s multiple comparisons test.

Cellular immune responses were evaluated in the lower airways and peripheral blood using *in vitro* antigen-recall assays. BAL mononuclear cells (Fig. 5A,B) and PBMC (Fig. 5C,D) were isolated on day 7 after BRSV infection and restimulated *in vitro* as described in Materials & Methods. Cell culture supernatants were then analyzed by sandwich ELISA for IL-17A (left panels) and IFNγ (right panels). BAL cells and PBMC from VAS/BRSV-NP vaccinated calves secreted IL-17A and IFNγ in response to BRSV restimulation and their response was significantly increased over both VAS/unvaccinated controls and over VAD/BRSV-NP vaccinated animals (Fig. 5). BAL and PBMC samples were also collected immediately prior to challenge and evaluated for vaccine-induced cytokine production. However, similar to the IgA results, we did not detect a vaccine-induced cellular response in either the airways or peripheral blood prior to BRSV challenge.

### Altered inflammatory cytokine expression in the respiratory tract of VAD calves

**Figure 6:**
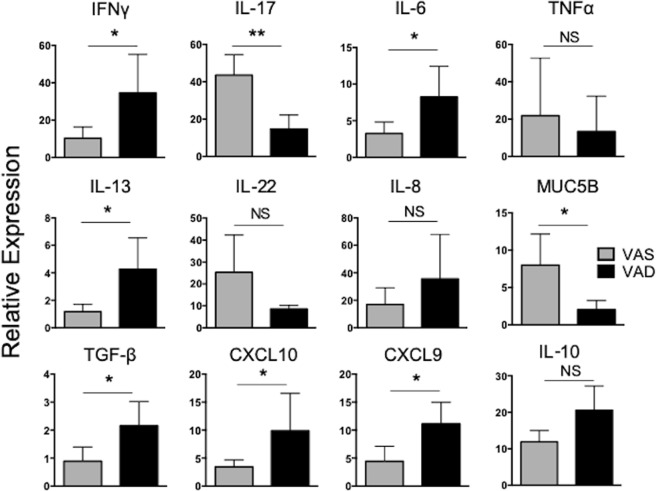
VAD significantly alters the lung inflammatory environment during BRSV infection. Lung samples were collected from unvaccinated, BRSV infected, VAS and VAD calves (n = 6 per group) on day 7 post infection and analyzed by qPCR for mRNA expression of the indicated gene targets. Results were normalized to the housekeeping gene RPS9 and expressed relative to lung samples from uninfected, VAS (n = 3) and VAD (n = 3) control calves. Data represent means ± SEM. *p < 0.05, **p < 0.01, as determined by student’s *t-*test.

VA has a significant impact on cells of the innate and adaptive immune system, particularly in the gastrointestinal tract^10,16,17^; however, less is understood about the impact of VAD on the immune system in the respiratory tract, particularly in the context of acute viral infection. In a mouse model of pneumovirus infection, VAD animals present with a ‘hyper-inflammatory’ phenotype in the lungs, with elevated expression of multiple proinflammatory cytokines^41,42^. To investigate the impact of VAD on BRSV infection, lung tissues samples were collected from unvaccinated, BRSV infected VAS and VAD calves on day 7 after infection and analyzed by qPCR for expression of proinflammatory cytokines and chemokines. As seen in Fig. 6, the expression of IFNγ, IL-6, IL-13, CXCL9, CXCL10 and TGF-β was significantly higher in the lungs of VAD animals compared to VAS controls. In contrast, the expression of IL-17 and the mucin gene, MUC5B, were both significantly reduced in VAD animals compared to VAS calves. The expression of TNFα, IL-22, IL-8, and IL-10 were not significantly different between VAD and VAS animals. Thus, VAD can enact profound alterations in the immune response during acute respiratory infection.

### Acute BRSV infection negatively impacts plasma and liver retinol in vitamin replete animals

**Figure 7:**
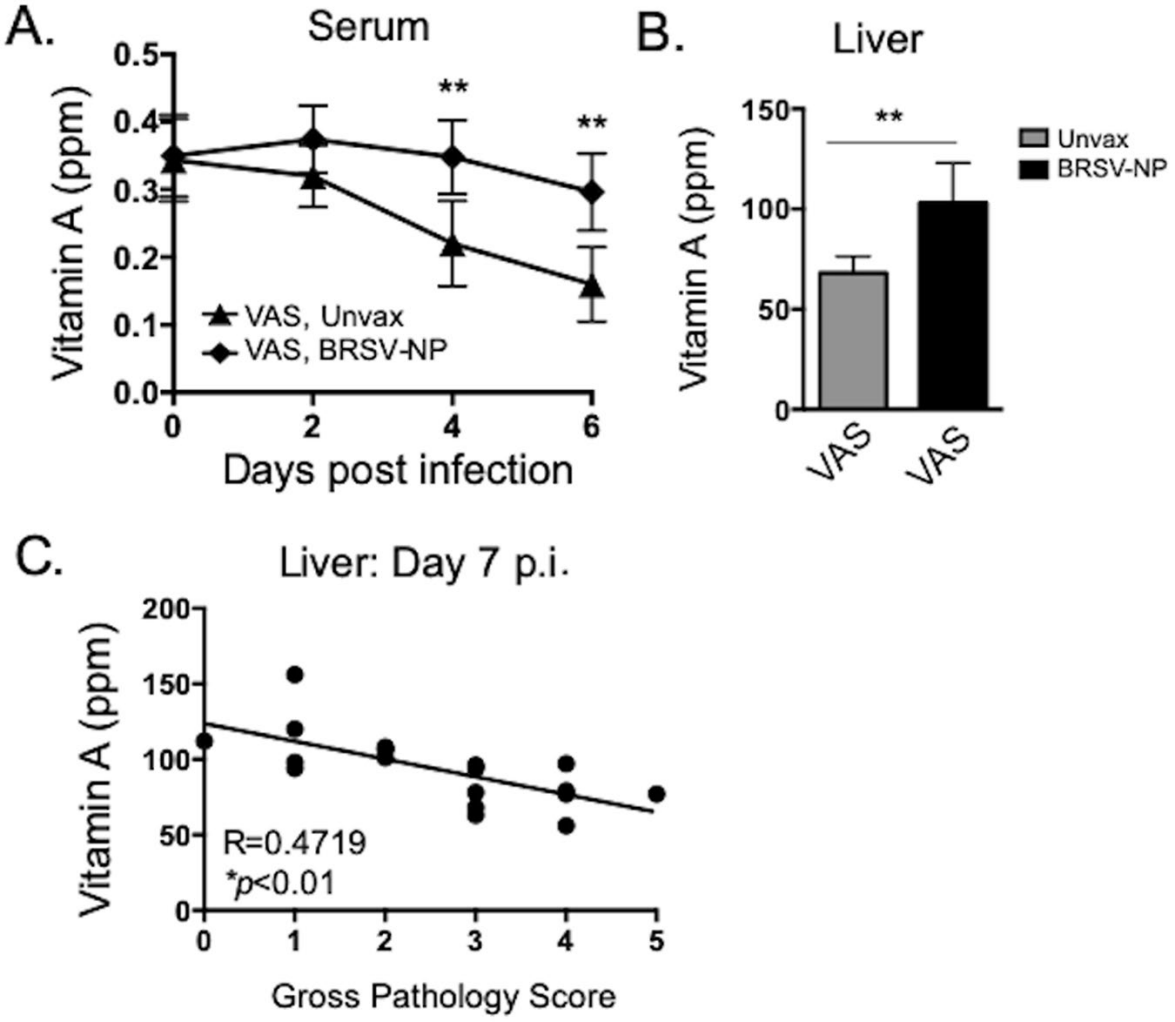
Acute BRSV infection negatively impacts VA status in vitamin-replete animals. (**A**) Serum retinol in VAS/BRSV-NP vaccinated and VAS unvaccinated calves on days 2, 4 and 6 after BRSV infection. **p < 0.01 by two-way ANOVA and Tukey’s post-test. (**B**) Liver retinol concentrations in VAS calves on day 7 after BRSV infection (as in Fig. 1B), grouped according to vaccination status (unvaccinated, grey bars; BRSV-NP vaccinated, black bars). **p < 0.01, as determined by student’s *t-*test. (**C**) Correlation plot comparing liver retinol concentrations from individual VAS calves (y-axis) with gross lung pathology scores (x-axis, as in Fig. 1B). Data were analyzed for correlation by nonparametric Spearman’s correlation test.

Acute illness leads to a sharp decrease in circulating retinol concentrations^43^, an outcome which has been termed hyporetinemia and is linked to the acute phase response. In rodents challenged with endotoxin, retinol is transiently sequestered in the liver and other tissues, but reappears in the plasma shortly after, with no appreciable irreversible retinol loss^44^. In human patients, however, acute illness has been linked to increased VA deficiency^43^. Observationally, respiratory infections in particular have been implicated in depletion of liver VA stores in children^45^; however, to our knowledge, there have been no studies to date investigating the impact of RSV infection on VA levels in nutritionally-replete infants. Given the potential link between acute infection and VA status, we more closely examined retinol levels in the VAS animals undergoing acute viral infection. Serum samples were collected from VAS/BRSV-NP vaccinated and VAS/unvaccinated calves on days 0, 2, 4 and 6 post infection and were analyzed for serum retinol. As seen in Fig. 7, consistent with reports of hyporetinemia in other species, serum VA levels declined in VAS/unvaccinated calves during acute BRSV infection. In contrast, VA levels were maintained in VAS/BRSV-NP immunized calves, which were undergoing a significantly reduced clinical disease (Fig. 2A). When liver VA levels from VAS calves (Fig. 1B) were further analyzed based on vaccination status, we observed that unvaccinated calves had reduced liver VA levels compared to the vaccinated cohort (Fig. 7B). In fact, the mean liver retinol levels fell below the accepted normal range for animals of this age^38^, rendering them VA deficient, despite their VA replete diet. We also analyzed liver retinol data from all VAS calves based on BRSV-associated lung pathology scores. As seen in Fig. 7C, liver retinol concentrations were negatively correlated with lung pathology (as an indicator of disease pathology). Thus, acute RSV infection has a significant negative impact on both circulating and stored retinol levels, even in vitamin-replete individuals.

## Discussion

Developing efficacious vaccines for RSV is challenging due to the nature of the virus, but also because the target population poses significant obstacles. RSV infection is most severe in premature infants, neonates and young children; and complications from RSV infection are most prevalent in developing countries^21,23^. VAD is endemic in the geographical regions which are hit hardest by RSV^1^, and is also a significant problem in premature infants in both developing and developed countries^7,8^. Also concerning, while VAD is thought to be a disease of the developing world, some studies have suggested that subclinical VAD may be quite prevalent^1,4,5,46^. It is critical to identify and address comorbidities and host factors which have potential to undermine the effectiveness of a vaccine, such as VAD, in target populations. Here, in the calf model of BRSV infection, we show that VAD has a profound negative impact on the capacity of the host to respond to an intranasal, polyanhydride NP vaccine and to resist the subsequent viral challenge. VAD calves have altered inflammatory cytokine profiles in the lungs, and do not produce virus-specific IgA or cellular immune responses in the lungs or peripheral blood. VA and RA play defined roles in gut immune function and homeostasis; however, our results emphasize that VA also plays an important role in immune function in the respiratory tract.

VAD calves demonstrated a heightened inflammatory cytokine profile in the lungs during BRSV infection, with increased expression of IFNγ, IL-13, IL-6 and the IFNγ-inducible chemokines CXCL9 and CXCL10. This result is consistent with a previous report by Penkert *et al*. in a mouse model of Sendai virus (SeV) infection^41^, where levels of IL-6, CXCL10, IFNγ and RANTES were increased in nasal washes from VAD, infected animals. In this model, similar to ours, VAD mice fail to produce virus-specific IgA and have reduced frequencies of CD8 T cells in the lungs following infection^42^. RA promotes the development of FoxP3^+^ regulatory T cells, while inhibiting Th17 cell differentiation^47^. The dysregulated hyper-inflammatory phenotype that has been observed in some models of VAD has been attributed to deficiencies in FoxP3^+^ Treg cells^48^. However, Penkert *et al*. showed that there is no defect in Treg cell differentiation in SeV-infected mice^41^. Given the reciprocal relationship between Treg and Th17 cells in the presence of RA^47^, one would predict increased Th17 development and increased IL-17 expression in a VAD system, thus explaining the altered inflammatory profile. However, this is not consistent with our findings in the lungs of VAD calves, as we observed significantly reduced expression of IL-17. Similarly, IL-17 was not detected in the nasal washes of VAD animals during SeV infection^41^. In a report by Cha *et al*., VAD mice have significantly reduced expression of IL-17 and reduced numbers of Th17 cells in the lamina propria^49^. Rudraraju *et al*. attributed the altered inflammatory profile in the lungs of SeV infected mice to increased viral antigen and viral persistence in the respiratory tract. When animals are sacrificed at the peak of the infection (day 7), the authors observe no difference in the viral titers between VAS and VAD animals^42^; however, on day 10 after infection, VAS animals had significantly reduced viral loads compared to VAD animals^41^. The authors speculate that antigen persistence and defects in viral clearance mechanisms contribute to the heightened inflammation in the lungs of the VAD animals, rather than defects in Tregs or Th17 skewing. We detected no differences in virus shedding or viral load between VAD or VAS calves on day 7 after infection. In future studies, it will be important to determine if VAD calves display similar defects in viral clearance in the later stages of the disease, thus explaining the dysregulated cytokine production we observed in the lungs, or if instead, this defect may be attributed to other mechanisms during RSV infection such as an imbalance in the Treg/Th17 axis. Importantly, we observed no significant differences in IL-10 expression in the lungs of VAD calves compared to VAS controls, suggesting that the Treg response may be intact in these animals. However, we did observe increased expression of TGF-β in the lungs of our VAD calves. While TGF-β expression can play a regulatory role, it is also an important cytokine that is elevated during during tissue repair and can contribute to lung fibrosis and mucin expression^50^. Further investigation into the impact of VAD on the respiratory immune system and response to RSV infection is warranted.

Nutritionists have long recognized that acute infection exacerbates the risk of malnutrition in children^43^. Measles, chickenpox, respiratory infections and diarrhea have all been implicated in the depletion of liver VA stores and increased risk of xerophthalmia, the principal clinical manifestation of VA deficiency^45,51^. However, there have been relatively few studies that have specifically addressed the impact of acute respiratory viral infection on retinol status in vitamin replete individuals. As we observed, serum retinol declines over the course of a severe BRSV infection. This result is not surprising, as serum VA is regulated during the acute phase response. In VA-adequate rats, serum retinol declines rapidly following LPS or IL-6 challenge, but returns to normal levels during the resolution phase of the response^44,52^. There is a minimal loss of retinol through the urine, and studies of radiolabeled retinol have revealed that the majority of retinol is redistributed in the liver and tissues during infection and returns to circulation^53^. However, contrary to these reports and consistent with the epidemiologic data from humans, our own data suggests that acute infection causes an overall loss in liver stores. In our studies, it is not clear if retinol stores are consumed during the inflammatory response or excreted during the illness. However, the severe impact of acute viral infection on VA stores is cause for concern, as there is potential to fall into a ‘vicious circle’ of acute illness, deficiency and susceptibility to secondary infection.

VA supplementation is an obvious solution to VAD. The WHO currently recommends large-dose, oral Vitamin A supplementation at immunization contact points^1^. VA supplementation has a significant positive impact on incidence of measles^54–56^. However, its effects on other diseases are less clear. Some studies have reported significant reductions, while others have found little effect or even increased incidence and severity of diarrhea and respiratory infections in children receiving vitamin A supplementation^36,57–60^. Studies specifically examining the impact of oral VA supplementation during RSV infection have shown no effect^36,57,61^ or in one study, have observed increased length of hospital stay for infants receiving VA^58^. We speculate that oral supplementation at the time of vaccination may not be ideal for addressing VAD and its effects on the respiratory immune response, primarily due to kinetics. A similar principle applies to supplementation during acute infection. Changes in VA homeostasis due to acute inflammation likely limits VA access to inflamed tissues and prevents any modulation to the ongoing immune response. Thus, in the future, we are interested in investigating the utility of a mucosal VA supplementation strategy, which may be a more efficient approach to addressing the immunologic lesions observed in VAD individuals during respiratory vaccination or infection.

In our previous studies we have shown that a single dose of the BRSV-NP vaccine affords partial protection against BRSV infection^34^. In some early studies in murine models, inclusion of a soluble protein bolus with NP encapsulated antigens has been used to boost the immunogenicity of the NP vaccine^29,39^. Therefore, in the current study, we hypothesized that administration of soluble F and G protein in suspension with the NP encapsulated proteins, would enhance efficacy. When comparing the vaccine-induced immune responses, viral burden and lung pathology from this study to our prior results^34^, we observed no increase in immunogenicity or protection mediated by this modified approach. This result is consistent with other recent reports from our groups, wherein a free protein component was not required for polyanhydride nanovaccine efficacy in a mouse model of influenza A virus^32^ or *Streptococcus pneumoniae*^62^ infection. In future studies, a heterologous prime-boost approach or use of additional RSV target proteins may prove more efficacious in the neonate.

In summary, we have confirmed our prior studies, showing that a single, intranasal dose of a BRSV-NP vaccine primes for protective immunity in neonatal calves, resulting in significant reductions in virus-associated clinical disease, lung pathology and viral lung burden following BRSV challenge. VAD enacts major changes in the lung inflammatory response during BRSV infection and has a severe negative effect on the immunogenicity of the mucosal BRSV-NP vaccine. We further show that acute RSV infection has the potential to induce or exacerbate VAD, even in well-nourished individuals, indicating that particular care should be taken in managing the nutrition of convalescent infants and children to avoid long-term micronutrient deficiencies and thus, susceptibility to secondary infections or complicating sequalae.

## Materials and Methods

### Recombinant F and G proteins

The recombinant fusion (F) protein was produced by Genscript (Piscataway, NJ) using a recombinant baculovirus expression system as we have previously described^34^. The recombinant attachment (G) protein was expressed in *E. coli* as we have previously described^34^.

### BRSV-F/G Nanovaccine

The BRSV-F/G nanovaccine was prepared as previously described^34^, with minor modifications. Briefly, 50:50 CPTEG:CPH copolymer was dissolved in methylene chloride at a concentration of 20 mg/mL. The F and G proteins were suspended in this solution at a total protein concentration of 7.4 mg/mL (3.7 mg/mL of each protein), sonicated for approximately 30 seconds, and the suspension poured into pentane at a 1:200 volumetric ratio (methylene chloride:pentane). The suspension was vacuum filtered to collect the nanoparticles and the nanoparticles were placed in a −20 °C freezer until further use. The nanoparticle yield was 73% with a protein encapsulation efficiency of ca. 100% ([actual protein mass encapsulated]/[theoretical protein mass encapsulated]).

### Animals

All animal procedures were conducted in strict accordance with federal and institutional guidelines and were approved by the Kansas State University Institutional Animal Care and Use Committee. The animal care protocol included provisions for a humane endpoint as determined by the discretion of the attending clinical veterinarian. Methods to minimize pain and distress included the avoidance of prolonged restraint and the inclusion of euthanasia as an intervention strategy.

A total of forty-three, mixed-gender (n = 21 males; n = 22 females) Holstein calves were purchased from two dairy farms, one in southern Nebraska (n = 37) and one in central KS (n = 6), and were enrolled in the study at birth. Calves were randomly assigned to the VAD or VAS treatment group. All calves were prevented suckling from their dams, and instead received a first feeding of colostrum replacer within 4 h of birth. Each animal received 375 g of fractionated colostrum replacer (Milk Products, Chilton, WI) reconstituted in 1.9 L of water at approximately 40 °C. The colostrum replacer contained 150 g of bovine globulin protein concentrated from colostral whey and was essentially devoid of all fat-soluble vitamins A, D_3_, and E. Vitamins D_3_ (150,000 IU of cholecalciferol/dose) and E (1,500 IU alpha-tocopheral/dose) were added back to the colostrum replacer for all calves. VA (150,000 IU retinyl palmitate) was added back to the VAS treatment group. VA was omitted from the colostrum replacer for the VAD calves. Animals were then placed on a VAS or VAD milk replacer diet for the remainder of the study (Milk Products, Chilton, WI). Calves were transported to the Large Animal Research Center at Kansas State University at 3–4 days of age and housed in groups of 2–3 calves per pen on pine chip bedding. Calves were bottle-fed three times per day (~8 hours apart) until 3 weeks of age, then twice per day (~12 hours apart). The milk replacer consisted of 21% crude protein and 20% fat, and was fed at a rate of 1.5 lbs/day and 14% solids. VAS calves received 45,000 IU VA (retinyl palmitate) per day. VAD animals received the same milk replacer formulation without VA. Calves were also provided ab libitum access to calf starter pellets (18% crude protein, 8% ADF) starting at ~2 weeks of age. Starter grain was formulated without added VA (VAD calves) or with 1820 IU/lb (VAS calves).

Serum retinol levels were monitored weekly. Samples were submitted to the Iowa State University Veterinary Diagnostic Laboratory for evaluation. At 6 weeks of age, animals were divided into 4 groups: 1) VAS, no vaccine (n = 6; 3 females and 3 males); 2) VAS, BRSV-NP vaccine (n = 13; 7 males and 6 females); 3) VAD, no vaccine (n = 6, 2 males and 4 females); 4) VAD, BRSV-NP vaccine (n = 12; 5 males and 7 females). Calves were vaccinated intranasally with 145 mg BRSV-F/G loaded CPTEG:CPH nanoparticles (~2 mg recombinant F and G proteins) with 2 mg soluble recombinant F and G proteins. The vaccine was suspended in 5 mL of sterile saline, with 2.5 mL delivered into each nostril. The calf was restrained and its head was tilted up, then the vaccine was administered into the nose using a syringe fitted with a 2-inch nasal cannula, equipped with a plastic depth control ring. Following vaccination, immune responses were monitored weekly in the serum, peripheral blood, and upper (nasal fluid collection) and lower (bronchoalveolar lavage fluid collection) respiratory tract. Six calves (VAS, n = 3, all calves were male; VAD, n = 3, 2 females and 1 male) were sacrificed prior to challenge and samples of lung and liver tissues were collected to verify retinol concentrations and to serve as control tissues for immunology and gene expression studies.

### Bronchoalveolar lavage fluid collection

Pre-challenge bronchoalveolar lavage (BAL) fluid was collected from all animals, 4–5 days prior to BRSV infection, using a protocol we have previously described^63^. A modified stallion catheter was blindly passed through the nose and advanced through the trachea until lodging in the bronchus. A total of 180 mL of sterile saline was divided into three aliquots. An aliquot was introduced to the lower respiratory tract, followed by immediate suction to obtain lower airway washes. The procedure was repeated twice more. All three aliquots were pooled at the end of the procedure. BAL samples were kept on ice, filtered over sterile gauze, and centrifuged at 200 x g for 10 minutes. Contaminating red blood cells were removed using hypotonic lysis. Cells were washed, counted and stimulated as described below for antigen recall assays.

### BRSV inoculum and aerosol challenge model

BRSV strain 375 was prepared from virus stock re-isolated from the lung of an infected animal and passaged less than 4 times on bovine turbinate (BT) cells. The viral inoculum was determined free of BVDV contamination by PCR. Calves were inoculated via aerosol challenge with ~10^4^ TCID_50_/mL of BRSV strain 375 as previously described^28^.

### Clinical Illness scoring

Calves were monitored twice daily for clinical illness by a trained, blinded observer. Calves were scored using an adaptation of the University of Wisconsin Calf Health Respiratory Scoring Chart, originally established by Dr. Sheila McGuirk (https://www.vetmed.wisc.edu/dms/fapm/fapmtools/8calf/calf_respiratory_scoring_chart.pdf). The scoring chart assigns numbers (0–3) based upon fever and severity of clinical signs including cough, nasal discharge, ocular discharge and ear position. For our scoring chart we include one additional category for respiratory effort (0 = no effort to 3 = significant effort).

### Virus isolation

Nasal swabs were collected from each calf on days 0, 3 and 7 post infection and placed in virus isolation media (serum free MEM with antibiotics). Virus isolations were performed as previously described^28^.

### Necropsy and pathological evaluation

Calves were euthanized on day 7 post-infection (p.i.) by barbiturate overdose. Pathological evaluation was performed similar to previous descriptions^28,64^. The extent of pneumonic consolidation was evaluated using the scoring system we have published^34^.

Post-infection BAL fluid was collected at necropsy by removing the lungs and trachea and introducing 500 mL of sterile, ice-cold saline through the trachea. The lungs were massaged, and then fluid was poured back out of the trachea into sterile collection bottles. Samples of affected and unaffected lungs were collected from multiple sites for histopathological evaluation. Tissues were fixed by immersion in 10% neutral buffered formalin and processed by routine paraffin-embedment and sectioning. 5 μM sections were stained for hematoxylin and eosin. Microscopic lesions were evaluated by a pathologist (Dr. Henningson) in a blinded manner. The severity of the lung lesions were scored based upon the criteria we have previously published^34^.

### Antigen recall assays

Peripheral blood mononuclear cells (PBMC) were prepared as previously described^34^. 5 × 10^6^ cells/mL were plated in round-bottom 96-well plates. Bronchoalveolar lavage fluid (BAL) samples were filtered through 2-layer sterile gauze and centrifuged a 500xg for 10 minutes. 10^6^ cells/well were resuspended in cRPMI and plated in 24 well plates. PBMC and BAL cells were stimulated with 5 μg/mL recombinant BRSV F or G protein or 0.01 MOI of BRSV. Plates were incubated for 6 days (PBMC) or 72 hours (BAL cells) at 37 °C in a 5% CO_2_ incubator. Cell culture supernatants were stored at −80 °C until ELISA analysis.

### Real-time PCR

RNA isolation, cDNA preparation and qPCR were performed as described^34^ using primer sequences which have been published^28^. Relative gene expression was determined using the 2^−ΔΔCt^ method^65^, with RPS9 as the reference housekeeping gene. Samples were normalized to uninfected control lung tissue collected from either VAS or VAD calves, as appropriate. NS2 qPCR was performed using the Taqman RNA-to-CT 1-step kit (Applied Biosystems) as in^34^. Viral NS2 copy numbers were calculated using standard curves. For lung tissue, NS2 copy numbers were normalized to the S9 housekeeping gene to correct for differences in input material. qPCR was run on an Agilent MX3000P Real-Time PCR machine or a ThermoFisher Scientific QuantStudio 3 Real-Time PCR machine.

### ELISAs

Bovine IL-17A and IFNγ VetSet ELISA Development kits were purchased from Kingfisher Biotech, Inc and performed according to manufacturer’s instructions.

Indirect ELISAs were used to quantify IgA and IgG in the nasal and BAL fluid. ELISA plates were coated overnight at 4 °C with 3 μg/mL F or G protein (IgA only), or with 100 μL/well of BRSV stock (~10^4^ TCID_50_). Negative control wells were coated with 100 μL/well cell culture media prepared from uninfected BT cells. Nasal fluid samples were diluted 1:2 and treated with 10 mM dithiothreitol (DTT) for one hour at 37 °C prior to performing the ELISAs. BAL fluid samples were diluted 1:2 with wash buffer. All samples were plated in duplicates, incubated for 2 h at room temperature and then washed. Mouse anti-bovine IgA-HRP (Bethyl Laboratories) or mouse anti-bovine IgG-HRP was used at 0.5 μg/mL. Plates were developed using Pierce 1-Step Ultra TMP Substrate (ThermoScientific Pierce). The reaction was stopped with the addition of 0.2 M H_2_SO_4_ and plates were read at an optical density of 450 nm and 540 nm using an automated plate reader.

### Serum neutralization assays

Baseline (prior to vaccination) and pre-challenge (day 0) serum samples were collected and submitted to the Kansas State University Veterinary Diagnostic Laboratory for evaluation of BRSV-specific virus neutralization titers.

### Statistics

Statistical analysis was performed using Prism v6.0 f software (Graphpad Software, Inc.). The data were analyzed using a Kruskal-Wallis test followed by Dunn’s Multiple Comparison’s test or by Student’s *t-*test. Correlation analysis in Fig. 7 was performed using Spearman’s nonparametric correlation test.

## Supplementary information

Supplemental Figure 1
