# Supplementary material for: Vitamin A deficiency impairs the immune response to intranasal vaccination and RSV infection in neonatal calves
Source: Sci Rep. 2019 Oct 22;9:15157. doi: 10.1038/s41598-019-51684-x (PMC6805856; doi:10.1038/s41598-019-51684-x)
Supplement: Supplementary file 1 — Supplemental Figure 1 [file 41598_2019_51684_MOESM1_ESM.pdf]

Vitamin A deficiency impairs the immune response to intranasal vaccination and RSV infection in neonatal calves

Jodi L. McGill[1,5], Sean M. Kelly[2], Mariana Guerra-Maupome[1], Emma Winkley[3], Jamie Henningson[3], Balaji Narasimhan[2,5], Randy E. Sacco[4,5]

[1]Department of Veterinary Microbiology and Preventative Medicine, Iowa State University, Ames, IA

[2]Department of Chemical and Biological Engineering, Iowa State University, Ames, IA

[3]Department of Diagnostic Medicine and Pathobiology, Kansas State University, Manhattan, KS

[4]Ruminant Diseases and Immunology Research Unit, National Animal Disease Center, Agricultural Research Service, USDA, Ames, IA

[5]Nanovaccine Institute, Iowa State University, Ames, IA

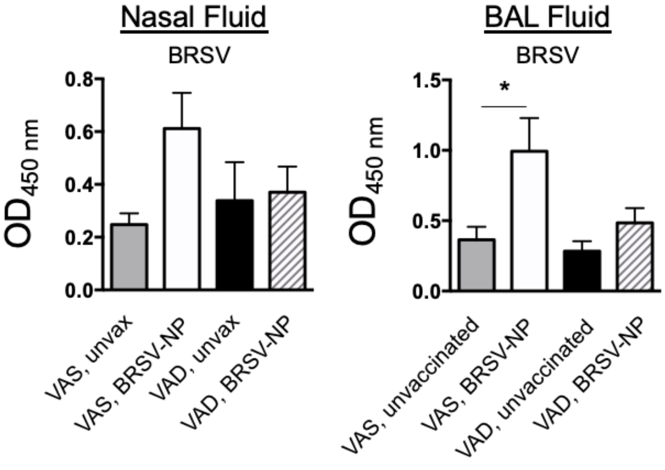

Supplementary Fig. 1. Virus-specific IgG in nasal secretions and BAL fluid of VAD and VAS animals. Nasal fluid and BAL samples were collected on day 7 post infection. The samples were diluted 1:10 and analyzed by indirect ELISA for BRSV-specific IgG. Data represent means ± SEM. *$p<0.05$ as determined by Kruskal-Wallis test, followed by Dunn's multiple comparisons test.
